# Enhanced adherence counselling and viral load suppression in HIV seropositive patients with an initial high viral load in Harare, Zimbabwe: Operational issues

**DOI:** 10.1371/journal.pone.0211326

**Published:** 2019-02-05

**Authors:** Talent Bvochora, Srinath Satyanarayana, Kudakwashe C. Takarinda, Hilda Bara, Prosper Chonzi, Brian Komtenza, Clemence Duri, Tsitsi Apollo

**Affiliations:** 1 City of Harare Health Services Department, Harare, Zimbabwe; 2 Centre for Operational Research, International Union Against Tuberculosis and Lung Disease, Paris, France; 3 Government of Zimbabwe, Ministry of Health and Child Care, AIDS and TB Unit, Harare, Zimbabwe; State of Israel Ministry of Health, ISRAEL

## Abstract

**Background:**

In people living with HIV (PLHIV) who are on anti-retroviral therapy (ART), it is essential to identify persons with high blood viral loads (VLs) (≥1000 copies/ml), provide enhanced adherence counselling (EAC) for 3 months and assess for VL suppression (<1000 copies/ml).

**Objective:**

Our study objectives were to determine the proportion who had a high viral load in those people who underwent viral load testing between 1 August 2016–31 July 2017 at Wilkins Hospital, Harare, Zimbabwe. Of those with high viral load to assess; a) the proportion who enrolled for EAC, the demographic and clinical characteristics associated with enrolment for EAC and, b) the proportion who achieved viral load suppression and demographic, clinical characteristics associated with viral load suppression.

**Design:**

Retrospective cohort study using routinely collected programme data. Data was collected from PLHIV who were on ART and had a high viral load from 1 August 2016 to 31 July 2017.

**Results:**

Of 5,573 PLHIV on ART between 1 August 2016 and 31 July 2017, 4787 (85.9%) had undergone VL testing and 646 (13.5%) had high VLs. Of these 646, only 489 (75.7%) were enrolled for EAC, of whom 444 (69%) underwent a repeat VL test at ≥ 3 months with 201 (31.2%) achieving VL suppression. The clinical characteristics that were independently associated with higher probability of VL suppression were: a) undergoing 3 sessions of EAC; b) being on 2^nd^ line ART. Initial VL levels >5,000 copies/ml were associated with lower probability of viral suppression.

**Conclusion:**

The routine VL testing levels were high, but there were major programmatic gaps in enrolling PLHIV with high VLs into EAC and achieving VL suppression. The full potential of EAC on achieving viral load suppression has not been achieved in this setting. The reasons for these gaps need to be assessed in future research studies and addressed by suitable changes in policies/practices.

## Introduction

Globally, since the beginning of the human immunodeficiency virus (HIV) epidemic in the 1980s, about 35 million people are estimated to have died due to HIV infection and by the end of 2016, an estimated 36.7 million [95% CI: 30.8–42.9 million] people were living with HIV (PLHIV) [1]. The burden of the HIV epidemic varies considerably in the world with 64% of the global HIV burden concentrated in sub-Saharan African countries.

In PLHIV, viral load (expressed as HIV RNA copies/mL of blood) is a direct indicator of viral replication. Higher viral loads lead to greater fall in CD4 cell count, and this increases the risk of becoming ill due to opportunistic infections [2]. Suppressing viral load in PLHIV to less than 1000 copies/ml of blood (henceforth called ‘viral suppression’) is essential for reducing morbidity, mortality and transmission [3].

Anti-retroviral therapy (ART) suppresses HIV replication and by doing so, it has transformed HIV infection from a deadly disease into a manageable chronic illness [2]. The recent HPTN052 clinical trial has shown that viral suppression due to ART can reduce HIV transmission by up to 96% [4]. In order to maximise the benefits of ART globally, the second and third targets of the Joint United Nations Programme for HIV/AIDS (UNAIDS) 90-90-90 target call on at least 90% of PLHIV to be on ART and 90% of those on ART to have viral suppression by 2020 [3].

World Health Organisation (WHO) currently recommends periodic assessment of viral loads (at least once a year) in all PLHIV on ART and to achieve viral load suppression in those with high plasma viral loads (≥1000 copies/ ml) by addressing the common reasons for it [2]. Poor adherence to ART is the most common reason for high viral load and, therefore, WHO recommends enhanced adherence counselling (EAC) to address this problem [5]. The other common reasons for high viral load include drug resistance, malabsorption, drug–drug interactions, drug-associated side effects and addressing these reasons may require a change in the ART regimen [6]. WHO recommends that, if the viral load is high, EAC be carried out, followed by a second/repeat viral load test after 3 months. If the viral load levels remains high, virological treatment failure is concluded and patient should have a switch in ART regimen. Studies have shown that EAC leads to viral suppression in over 70% of patients with high initial viral loads [7].

Zimbabwe, a country in Southern Africa with a population of approximately 13 million people in 2012 [8], is one of the country worst affected by the HIV epidemic [9]. The HIV prevalence among adults 15–64 years is 14.6% which estimates to approximately 1.2 million PLHIV. About 75% of the PLHIV know their HIV status, and 86.6% of those who know their status are on ART. It is estimated that 15% of those on ART have high viral load [10]. The Zimbabwe National ART programme guidelines were adopted from the 2016 WHO guidelines and recommends management of persons with high viral load through EAC, adherence-monitoring, followed by a repeat viral load test and subsequently a switch in ART regimen if the viral load remains high [2]. However, whether these guidelines are being followed or not under routine program conditions is unknown. Therefore, we undertook an operational research study at Wilkins Hospital which was the first site to start routine viral load monitoring in Harare city health department, to assess progress in the implementation of the national guidelines.

Our study objectives were to determine the proportion who had a high viral load in those people who underwent viral load testing between 1 August 2016–31 July 2017. Of those with high viral load we assessed a) the proportion who enrolled for EAC, the demographic and clinical characteristics associated with enrolment for EAC and, b) the proportion who achieved viral load suppression and the demographic, clinical characteristics associated with viral load suppression.

## Methodology

### Study design

This was a retrospective cohort study using routine data of PLHIV who were on ART.

### Setting

The study was conducted at Wilkins Hospital, Harare city from October 2017 to March, 2018. As per the national guidelines, the ART clinic at Wilkins hospital uses a fixed-dose combination once-daily pill of Tenofovir v+ Lamuvidine (or Emtricitabine) + Efavirenz (TDF+3TC (or FTC)+EFV) as the preferred first-line ART regimen among adult PLHIV and abacavir+lamuvidine+efavirenz (or Lopinavir/r) (ABC + 3TC + EFV (or Lop/r)) as the preferred first-line ART regimen in children living with HIV. Second-line ART in adults consists of two nucleoside reverse-transcriptase inhibitors (NRTIs) plus a ritonavir-boosted protease inhibitor (PI). The choice of NRTI depends on the drug intake history. If the patient fails on a TDF + 3TC (or FTC)-based first-line regimen, AZT + 3TC is used as the NRTI backbone in second-line regimens and vice-versa in combination with a heat-stable fixed-dose combinations of ATV/r or LPV/r boosted PI.

The ART clinic of this hospital started conducting routine viral load testing of all PLHIV (either on first line regimen or second line regimen) from 1 August 2016 onwards as per the national guidelines. The national guidelines stipulate that PLHIV who do not have an initial viral load and are on ART for more than six months be tested for viral load. Viral load tests are also conducted to confirm treatment failure if there is a clinical deterioration. Those with viral load ≥1000 copies/ml are referred for enrolment for EAC. EAC consists of three sessions done on a monthly basis. After three EAC sessions, each client is assessed for adherence and a repeat viral load test is done. If the viral load is supressed (< 1000 copies/ml of blood), the client is continued on the same ART regimen. On the other hand, if the repeat viral load is high (≥ 1000 copies/ml of blood) despite good adherence to therapy, the client is referred to the ART medical officer for a change in the ART regimen. Children (age <15 years) with high viral load are also enroled for EAC and their adult care-givers are counselled on the importance of adherence.

### Patient population

The study population comprised of PLHIV who were on ART and had a high viral load during the period 1 August 2016 to 31 July 2017.

### Data variables, sources of data and data collection

For the first objective, we collected aggregated data on the number of patients who were currently alive and receiving ART from the electronic patient monitoring system (EPMS). We obtained information on the number who underwent viral load testing from the laboratory records of the hospital. We then line-listed all patients with high viral loads from the laboratory records and obtained individual client level information from the ART registers, individual patients ART booklets and EAC registers. The individual patient level information obtained included ART number, name, age, sex, date of HIV diagnosis, date of ART enrolment, date of ART initiation, dates of viral load testing, reason for doing viral load testing (routine or for confirmation of treatment failure) ART regimen, CD4 cell count levels, WHO stage, presence/absence of TB disease, cryptococcal co-infection at the time of initial high viral load, EAC enrolment and the number of EAC sessions attended. By using the date of ART initiation and last clinic visit date we derived the duration on ART. We also collected information on whether the patients had undergone repeat viral load testing, the results of the repeat viral load testing and whether the patient had undergone change in the ART regimen.

### Data analysis

Data from the ART registers, laboratory records and EAC registers were single-entered into an electronic data collection form created in EpiData Entry (version v2.2.3.187, The EpiData Association, Denmark). The data entered in EpiData was cross-checked with client information in the EPMS and wherever there was a discrepancy we referred to the original records and made the corrections. The data are summarized by numbers and proportions.

The primary outcomes were: a) “enrolment into EAC” which was defined as attendance of one or more EAC sessions, and, b) “viral load suppression” defined as viral load <1000 copies/mL at the time of repeat viral load test which is generally performed at/after 3 month. The primary focus of our analysis was in identifying which of the measured demographic and clinical characteristics are independently associated (predictors) with “enrolment into EAC” and for “viral load suppression”. The associations between measured demographic and clinical factors with “enrolment into EAC” and “viral load suppression” are expressed as crude/unadjusted risk ratios and adjusted risk ratios. We adjusted for all those variables that were previously known to be associated with the outcome or those which had a p-value of <0.2 in the bivariable analysis. We used multivariable binomial log regression models (or Poisson regression models if the log binomial models failed to achieve convergence with robust standard error estimates) to obtain the risk ratios and adjusted risk ratios. Due to high missingness of data and in order to retain the whole eligible number of records with an outcome of interest for the binomial log regression models, all data points with missing data were replaced with an arbitrary value “9” and reported in this manuscript as “Not recorded”. for each variable All analyses were done in STATA statistical software (SE version 15.0, StataCorp, Texas, USA).

### Ethics approval

We obtained approval from the National AIDS and Tuberculosis Unit and Harare City Research Board for conducting this study. We also obtained ethics approval from Medical Research Council of Zimbabwe (MRCZ) and the Ethics Advisory Group of The Union, Paris, France. As the study involved review of records, we were given waiver by the ethics committees from obtaining informed consent from the study participants. All data collected in this study were kept confidential and only the study investigators had access to the individual patient data.

## Results

**Fig 1:**
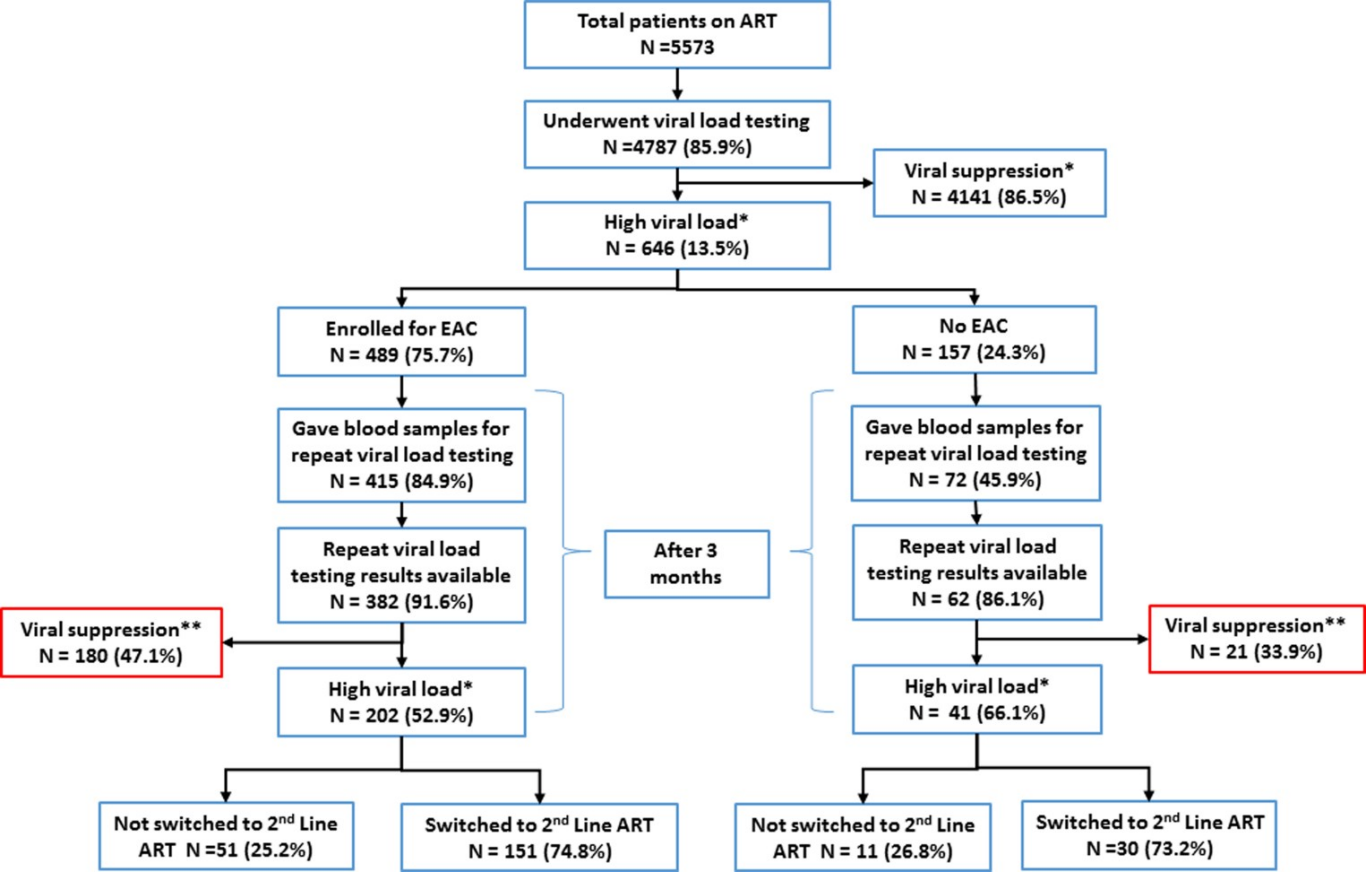
Management of people living with HIV on ART with high viral load ≥1000 copies/ml at Wilkin’s Hospital, Harare, 1 August 2016–31 July 2017.

There were 5,573 PLHIV who were alive and receiving ART care at Wilkin’s hospital between 1 August 2016 and 31 July 2017. Of these, 4787 (85.9%) had undergone viral load testing at least once during the study period. Of those tested, 646 (13.5%) had high viral loads “Fig 1”. The demographic and clinical characteristics of these 646 PLHIV with high viral loads are described in Table 1.

**Table 1:** Demographic and clinical characteristics of HIV-infected patients on ART with high viral load (≥1000 copies/ml of blood) at Wilkins Infectious Diseases Hospital, Harare (August 2016 –July 2017).

Characteristic	N	(%)
**Total**	**646**	**(100)**
**Gender**		
Female	355	(55.0)
Male	290	(44.9)
Not recorded	1	(<1)
**Age in years**		
<10	48	(7.4)
10–19	85	(13.2)
20–29	87	(13.5)
30–39	136	(21.1)
≥40	283	(43.8)
Not recorded	7	(1.1)
Median (IQR)	440	38 (22–45)
**Reason for VL test**		
Routine VL	584	(90.4)
Confirmation of treatment failure	55	(8.5)
Not recorded	7	(1.1)
**Duration on ART**		
0–2 years	122	(18.9)
3–5 years	239	(37.0)
6–10 years	217	(33.6)
Missing	68	(10.5)
Median (IQR)	578	4.9 (3.3–7.6)
**Last WHO stage**		
1 or 2	364	(56.4)
3 or 4	257	(39.8)
Not recorded	25	(3.9)
**TB co-infection**		
No	623	(96.4)
Yes	7	(1.1)
Not recorded	16	(2.5)
**Last CD4 cell count**		
≤200	148	(22.9)
201–350	98	(15.2)
>350	195	(30.2)
Not recorded	205	(31.7)
Median (IQR)	441	313 (146–509)
**First Viral load**		
1000–5000	216	(33.4)
5001–50 000	260	(40.3)
>50 000	170	(26.3)
**ART regimen**		
1^st^ line	458	(70.9)
2^nd^ line	181	(28.0)
Not recorded	7	(1.1)

HIV = Human Immunodeficiency Virus; ART = antiretroviral therapy; WHO = World Health Organisation;; TB = Tuberculosis; EAC = enhanced adherence counselling;

Of the 646 PLHIV with high viral loads, 489 (75.7%) were enrolled for EAC “Fig 1”. Of those enrolled for EAC, 30 (6%) attended one, 54 (11%) attended two and 405 (83%) attended three EAC sessions. Blood samples were collected for repeat viral load testing at or after 3 months in 487 (84.9% among those who underwent EAC compared to 45.9% in those who did not undergo EAC, Chi-square test p-value <0.01). In those whose blood samples were collected for repeat viral load testing, results were available for 444 (91.1%). Of those 444 with repeat viral load test results, 201 (45.2%) had viral load suppression (47.1% in those who underwent EAC and 33.9% in those who did not undergo EAC, Chi-square test p-value = 0.05).

### Patient characteristics associated with enrolment for EAC (Table 2)

In bivariable analysis, age, reason for viral load test, WHO stage, CD4 cell count, initial viral load levels were statistically associated with enrolment to EAC, but none of them were independently associated with enrolment for EAC in multivariable analysis.

**Table 2:** Demographic and clinical characteristics associated with enrolment for EAC sessions in HIV-infected patients on ART with high viral loads (≥1000 copies/ml of blood) at Wilkins Infectious Diseases Hospital, Harare (August 2016 –July 2017).

Characteristic	Total	EAC session done	RR (95% CI)	ARR (95% CI)
	N	n (%)		
**Total**	**646**	**489 (75.7)**	**-**	**-**
**Gender**				
Female	355	275 (77.5)	reference	reference
Male	290	213 (73.5)	0.95 (0.87–1.04)	0.99 (0.91–1.08)
Missing	1	1 (100)	-	-
**Age in years**				
30–39	136	94 (69.1)	reference	reference
<10	48	41 (85.4)	1.24 (1.05–1.45)	1.16 (0.98–1.36)
10–19	85	60 (70.6)	1.02 (0.86–1.22)	1.00 (0.84–1.18)
20–29	87	65 (74.7)	1.08 (0.92–1.28)	1.13 (0.98–1.30)
≥40	283	225 (79.5)	1.15 (1.01–1.31)	1.12 (1.00–1.26)
Missing	7	4 (57.1)	0.83 (0.43–1.59)	1.52 (0.69–3.36)
**Reason for VL test**				
routine VL	584	451 (77.2)	reference	reference
confirmation of treatment failure	55	34 (61.8)	0.80 (0.65–0.99)	0.94 (0.74–1.20)
not recorded	7	4 (57.1)	0.74 (0.39–1.41)	0.76 (0.15–3.89)
**Duration on ART**				
0–2 years	122	93 (76.2)	reference	reference
3–5 years	239	198 (82.9)	1.08 (0.97–1.20)	1.06 (0.94–1.19)
6–10 years	217	178 (82.0)	1.07 (0.96–1.19)	1.02 (0.90–1.15)
Missing	68	20 (29.4)	-	-
**Last WHO stage**				
1 or 2	364	281 (77.2)	reference	reference
3 or 4	257	195 (75.9)	0.98 (0.90–1.07)	1.02 (0.93–1.11)
not recorded	25	13 (52.0)	-	-
**TB co-infection**				
No	623	475 (76.2)	reference	reference
Yes	7	5 (71.4)	0.94 (0.59–1.50)	0.82 (0.47–1.42)
not recorded	16	9 (56.3)	-	-
**Last CD4 cell count**				
≤200	148	101 (68.2)	reference	reference
201–350	98	74 (75.5)	1.11 (0.95–1.30)	1.01 (0.87–1.17)
>350	195	170 (87.2)	1.28 (1.13–1.44)	1.14 (1.02–1.28)
not recorded	205	144 (70.2)	-	-
**First Viral load**				
1000–5000	216	182 (84.3)	reference	reference
5001–50 000	260	193 (74.2)	0.88 (0.80–0.97)	0.95 (0.87–1.03)
>50 000	170	114 (67.1)	0.80 (0.71–0.90)	0.91 (0.81–1.02)
**ART regimen**				
1^st^ line	458	340 (74.2)	reference	reference
2^nd^ line	181	145 (80.1)	1.08 (0.99–1.18)	1.05 (0.96–1.14)
Not recorded	7	4 (57.1)	-	-

HIV = Human Immunodeficiency Virus; ART = antiretroviral therapy; WHO = World Health Organisation; IQR = inter-quartile range; TB = Tuberculosis; EAC = enhanced adherence counselling; RR = relative risk; ARR = multivariate-adjusted relative risk; CI = confidence interval

### Patient characteristics associated with viral load suppression (Tables 3 and 4)

Overall of the 646 PLHIV with initial high viral load, 201 (31.2%) had viral load suppression on repeat viral load testing. The characteristics that were independently associated with higher probability of viral suppression were: a) undergoing 3 sessions of EAC compared to non-attendance of EAC; b) being on 2^nd^ line versus being on 1^st^ line ART at the time of initial high viral load test. Initial viral load levels >5,000 copies/ml were associated with lower probability of viral suppression when compared to having viral loads between 1000–5,000 copies/ml “Table 3”.

**Table 3:** Demographic & clinical characteristics associated with suppression of viral load on repeat testing in HIV-infected patients on ART with high initial viral loads (≥1000 copies/ml of blood) at Wilkins Infectious Diseases Hospital, Harare (August 2016 –July 2017).

Characteristic	Total	Repeat viral load<1000 copies/ml	RR (95% CI)	ARR (95% CI)
	N	n (%)		
**Total**	**646**	**201 (31.11)**	-	-
**Gender**				
Female	355	124 (34.9)	reference	reference
Male	290	76 (26.2)	0.75 (0.59–0.95)	0.91 (0.73–1.15)
Missing	1	1 (100)	-	-
**Age in years**				
30–39	136	94 (69.1)	reference	reference
<10	48	41 (85.4)	0.86 (0.51–1.45)	0.78 (0.47–1.28)
10–19	85	60 (70.6)	0.67 (0.41–1.08)	0.79 (0.51–1.23)
20–29	87	65 (74.7)	0.73 (0.46–1.15)	0.79 (0.53–1.18)
≥40	283	225 (79.5)	1.18 (0.89–1.58)	0.98 (0.75–1.28)
Missing	7	4 (57.1)	-	-
**Reason for VL test**				
routine VL	584	451 (77.2)	reference	reference
confirmation of treatment failure	55	34 (61.8)	0.68 (0.41–1.14)	1.47 (0.81–2.66)
not recorded	7	4 (57.1)	-	-
**EAC sessions**				
0	157	21 (13.4)	reference	reference
1–2	84	31 (36.9)	2.76 (1.70–4.49)	1.42 (0.88–2.29)
3	405	149 (36.8)	2.75 (1.81–4.18)	1.68 (1.09–2.58)
**Duration on ART**				
0–2 years	122	40 (32.8)	reference	reference
3–5 years	239	75 (31.4)	0.96 (0.70–1.31)	0.87 (0.65–1.15)
6–10 years	217	82 (37.8)	1.15 (0.85–1.57)	0.85 (0.63–1.15)
Missing	68	4 (5.9)	-	-
**Last WHO stage**				
1 or 2	364	281 (77.2)	reference	reference
3 or 4	257	195 (75.9)	1.01 (0.80–1.28)	1.03 (0.82–1.29)
not recorded	25	13 (52.0)	-	-
**TB co-infection**				
No	623	475 (76.2)	reference	reference
Yes	7	5 (71.4)	1.36 (0.57–3.23)	0.88 (0.22–3.52)
not recorded	16	9 (56.3)	-	-
**Last CD4 cell count**				
≤200	148	101 (68.2)	reference	reference
201–350	98	74 (75.5)	0.80 (0.49–1.31)	0.74 (0.47–1.18)
>350	195	170 (87.2)	1.96 (1.42–2.7)	1.49 (1.11–2.00)
not recorded	205	144 (70.2)	-	-
**First Viral load**				
1000–5000	216	182 (84.3)	reference	reference
5001–50 000	260	193 (74.2)	0.38 (0.29–0.5)	0.46 (0.35–0.6)
>50 000	170	114 (67.1)	0.33 (0.23–0.46)	0.4 (0.28–0.57)
**ART regimen**				
1^st^ line	458	340 (74.2)	reference	reference
2^nd^ line	181	145 (80.1)	1.65 (1.32–2.07)	1.54 (1.21–1.96)
Not recorded	7	4 (57.1)	-	-

HIV = Human Immunodeficiency Virus; ART = antiretroviral therapy; WHO = World Health Organisation; IQR = inter-quartile range; TB = Tuberculosis; EAC = enhanced adherence counselling; RR = relative risk; ARR = multivariate-adjusted relative risk; CI = confidence interval

Since ascertainment of viral load suppression is dependent on PLHIV having a repeat viral load test, we assessed the patient characteristics associated with viral load suppression in only those PLHIV who had a repeat viral load test result (n = 444). The demographic and clinical factors that were independently associated with higher probability of viral suppression in this sub-group were: a) CD4 cell count >350 cells/mm^3^; b) being on 2nd line ART regimen at the time of initial high viral load test result. Those with initial viral load levels >5000 copies per ml had a lower probability of viral load suppression when compared to those between 1000–5000 copies per ml “Table 4”. Enrolment for EAC (and the number of sessions attended) did not have an independent association with viral load suppression in this sub-group of PLHIV who had undergone repeat viral load testing.

**Table 4:** Demographic & clinical characteristics associated with suppression of viral load in those who underwent repeat viral load testing in HIV-infected patients on ART with high initial viral loads (≥1000 copies/ml of blood) at Wilkins Infectious Diseases Hospital, Harare (August 2016 –July 2017).

Characteristic	Total	Repeat viral load<1000 copies/ml	RR (95% CI)	ARR (95% CI)
	N	n (%)		
**Total**	**444**	**201 (45.3)**	**-**	**-**
**Gender**				
Female	245	124 (50.6)	reference	reference
Male	198	76 (38.4)	0.76 (0.61–0.94)	0.90 (0.73–1.11)
Missing	1	1 (100)	-	-
**Age in years**				
30–39	86	43 (50.0)	reference	reference
<10	38	13 (34.2)	0.68 (0.42–1.12)	0.73 (0.46–1.14)
10–19	53	18 (34.0)	0.68 (0.44–1.05)	0.79 (0.53–1.16)
20–29	59	20 (33.9)	0.68 (0.45–1.03)	0.76 (0.53–1.09)
≥40	204	106 (52.0)	1.04 (0.81–1.33)	1.00 (0.78–1.28)
Missing	4	1 (25)	-	-
**Duration on ART**				
0–2 years	85	40 (47.1)	reference	reference
3–5 years	179	75 (41.9)	0.89 (0.67–1.18)	0.87 (0.68–1.11)
6–10 years	168	82 (48.8)	1.04 (0.79–1.36)	0.78 (0.60–1.02)
Missing	12	4 (33.3)	-	-
**Reason for VL test**				
routine VL	420	187 (44.5)	reference	reference
confirmation of treatment failure	20	12 (60.0)	1.35 (0.93–1.96)	1.79 (1.05–3.03)
not recorded	4	2 (50.0)	-	-
**EAC sessions**				
0	62	21 (33.9)	reference	reference
1–2	61	31 (50.8)	1.5 (0.98–2.3)	0.95 (0.62–1.46)
3	321	149 (46.4)	1.37 (0.95–1.98)	1.11 (0.75–1.64)
**Last WHO stage**				
1 or 2	255	116 (45.5)	reference	reference
3 or 4	178	83 (46.6)	1.03 (0.83–1.26)	1.01 (0.82–1.23)
not recorded	11	2 (18.2)	-	-
**TB co-infection**				
No	431	196 (45.5)	reference	reference
Yes	4	3 (75.0)	1.65 (0.93–2.93)	1.63 (0.93–2.84)
not recorded	9	2 (22.2)	-	-
**Last CD4 cell count**				
≤200	98	36 (36.7)	reference	reference
201–350	68	19 (27.9)	0.76 (0.48–1.21)	0.83 (0.54–1.29)
>350	138	93 (67.4)	1.83 (1.38–2.44)	1.62 (1.23–2.15)
not recorded	140	53 (37.9)	-	-
**First Viral load**				
1000–5000	169	117 (69.2)	reference	reference
5001–50 000	171	54 (31.6)	0.46 (0.36–0.58)	2.22 (1.59–3.10)
>50 000	104	30 (28.9)	0.42 (0.30–0.57)	1.05 (0.73–1.51)
**ART regimen**				
1^st^ line	306	121 (39.5)	reference	reference
2^nd^ line	135	79 (58.5)	1.48 (1.21–1.8)	1.49 (1.2–1.84)
Not recorded	3	1 (33.3)	-	-

HIV = Human Immunodeficiency Virus; ART = antiretroviral therapy; WHO = World Health Organisation; IQR = inter-quartile range; TB = Tuberculosis; EAC = enhanced adherence counselling; RR = relative risk; ARR = multivariate-adjusted relative risk; CI = confidence interval

## Discussion

This is one of the first studies from Zimbabwe assessing the management of PLHIV on ART with high viral load under routine programme settings. The study results show that 86% of the PLHIV on ART at the Wilkin’s hospital had undergone a viral load test, 14% of those who underwent a viral load test had high viral loads which is similar to other studies [11]. Of those with high viral loads, three quarters had enrolled for EAC, two thirds had undergone a repeat viral load testing at 3 months (or later) and only about one third had achieved viral load suppression. Of those without viral load suppression at 3 months (or later), about three quarters had a change in ART regimen. The implications of the study are as follows:

First, 86% of the patients had undergone a viral load test and 14% had high viral loads. This implies that out of all the patients who were on ART at this study site, only 74% had a ‘confirmed’ viral load suppression. This is far below the 90% target for viral load suppression envisaged under the UNAIDS 90-90-90 targets [3]. For increasing the proportion with ‘confirmed’ viral load suppression to 90%, viral load testing levels must increase from the present 86%. The National AIDS programme must investigate if there are any local constraints for conducting viral load tests (e.g., in commodities, training needs, patient factors) and undertake appropriate measures to improve the testing levels.

Second, three-quarters of the patients with high viral load levels got enrolled into EAC and of those enrolled four out of five patients attended all the three sessions. Which means that of those PLHIV with high viral load, only 63% (~6 out of 10 patients with high viral load) underwent EAC as per the national guidelines. There is a potential threat that the remaining four in ten patients with high viral loads may develop adverse ART treatment outcomes [12] or of reversing the potential ART gains of reduced HIV transmission in the community [13]. As per our study results, none of the routinely recorded demographic and clinical characteristics were independently associated with enrolment into EAC. The other patient and health system constraints for enroling and retaining PLHIV on EAC are unknown. Identifying the constraints in our setting is an area for future research. Anecdotal evidence points towards deficiencies in the referral process from the clinicians responsible for their clinic reviews to primary care counsellors who offer EAC.

Third, of those with high viral loads, about two thirds (69%) underwent repeat viral load testing at 3 months or later. Patients who attended EAC were more likely to undergo the repeat viral load testing when compared to those who did not enrol for EAC. A previous study has shown that those who are adherent are more likely to undergo repeat VL testing [14].

Fourth, only about one third of the study population (with high initial viral loads) achieved viral suppression at 3 or more months. This is much lower than viral suppression rates reported elsewhere [7]. Grossly, those who underwent EAC were more likely to achieve viral load suppression than those who did not undergo EAC “Fig 1 and Table 3”. However in our subsequent analysis, in which we restricted our analysis to those who had undergone repeat viral load testing “Table 4”, we did not observe EAC as one of the factors associated with viral load suppression and this result is similar with other studies [6]. This indicates that EAC has limited effect on improving adherence in our setting or that the high viral loads seen in our settings is not due to poor adherence. Further studies are needed to sort this out. As a first step, we recommend a content analysis of the EAC counselling sessions to assess if these sessions are appropriate for identifying and correcting adherence related issues.

Lastly, being on 2^nd^ line ART (when compared to being on 1^st^ line ART) was independently associated with viral suppression. This could be due to poor initial adherence levels which was perhaps corrected by enrolment into EAC [15]. In addition, lower levels of resistance to 2^nd^ line ART (as shown in previous studies) [16] might have also contributed to the better response to therapy [12]. The other clinical factor that was independently associated with viral suppression was having viral load levels between (1000 to 5000 copies/ml) when compared to those with viral load >5000 copies/ml. Previous studies from other parts of the world show that initial viral load levels may be a good predictor of virological failure in patients with high adherence levels.[17] (i.e., patients with high baseline adherence levels may not benefit from EAC). Future studies in our setting could consider capturing information on baseline adherence levels and assess the effectiveness of EAC in supressing viral load in patients with different baseline adherence levels.

The major strength of the study was usage of routine programme data over a period of one year with information collected on all patients who were on ART at this hospital without any exclusion. We collected data from the records which are the first/primary level of documentation of the patient information in this setting. These primary records are routinely audited by the National AIDS programme for its accuracy and consistency. Therefore, we strongly believe that the data collected in our study reflects what is happening in reality in this setting.

The major limitation of our study is that as our study methodology involved review of records, and hence our analysis and interpretation of the data are limited to only those variables that are routinely collected from patients/care givers and captured in the patient records. Some of the important variables like socio-economic status of the patient, education status of the patient, distance of patients’ residence to the ART centres, patients clinical condition and so on, which could have played a major role in initial viral load testing, enrolment for EAC, repeat viral load testing and viral suppression, were not available. Since we did not measure these other variables, we are unable to account for the influence of these factors in our analysis. Therefore, whether the association estimates for demographic and clinical characteristics with the key outcome variables presented in our study is an underestimate or an overestimate is unknown.

In conclusion, at Wilkin’s hospital in Harare, about 14% of the patients who underwent viral load testing had high viral loads and only one third of these patients had viral load suppression at 3 months or later. The factors that were statistically associated with viral load suppression on repeat testing at 3 or more months were enrolment to EAC, having relatively lower levels of viral load and being on 2^nd^ line ART regimen. The study highlights several gaps in routine viral load testing, enrolment into EAC & repeat viral load testing. Due to these gaps, the role of EAC in achieving viral load suppression under routine programmatic conditions appears to be very limited. The reasons for these gaps needs to be assessed in future research studies and addressed by suitable changes in policies/practices.

## References

[1] UNAIDS. UNAIDS HIV data [Internet]. 2016 [cited 2017 Jul 14]. Available from: http://www.unaids.org/en/resources/fact-sheet

[2] World Health Organisation. WHO 2016 HIV guidelines. 2016 [cited 2017 Jul 13];1–259. Available from: http://apps.who.int/iris/bitstream/10665/208825/1/9789241549684_eng.pdf

[3] USAIDS. 90-90-90 An ambitious treatment target to help end the AIDS epidemic [Internet]. 2017 [cited 2017 Jul 15]. Available from: http://www.unaids.org/sites/default/files/media_asset/90-90-90_en.pdf

[4] Cohen MS, Chen YQ, McCauley M, Gamble T, Hosseinipour MC, Kumarasamy N, et al Antiretroviral Therapy for the Prevention of HIV-1 Transmission. N Engl J Med [Internet]. 2016 9 18 [cited 2017 Jul 15];375(9):830–9. Available from: http://www.nejm.org/doi/10.1056/NEJMoa1600693 27424812 10.1056/NEJMoa1600693 PMC5049503

[5] Fox MP, Cutsem G Van, Giddy J, Maskew M, Keiser O, Prozesky H, et al Rates and predictors of failure of first-line antiretroviral therapy and switch to second-line ART in South Africa. J Acquir Immune Defic Syndr [Internet]. 2012 8 1 [cited 2017 Jul 13];60(4):428–37. Available from: http://content.wkhealth.com/linkback/openurl?sid=WKPTLP:landingpage&an=00126334-201208010-00013 10.1097/QAI.0b013e3182557785 22433846 PMC3392418

[6] Jobanputra K, Parker LA, Azih C, Okello V, Maphalala G, Kershberger B, et al Factors Associated with Virological Failure and Suppression after Enhanced Adherence Counselling, in Children, Adolescents and Adults on Antiretroviral Therapy for HIV in Swaziland. Paraskevis D, editor. PLoS One [Internet]. 2015 2 19 [cited 2017 Jul 13];10(2):e0116144 Available from: http://dx.plos.org/10.1371/journal.pone.0116144 10.1371/journal.pone.0116144 25695494 PMC4335028

[7] Bonner K;, Mezochow A;, Roberts T;, Ford N;, Cohn J, Bonner K, et al Viral Load Monitoring as a Tool to Reinforce Adherence: A Systematic Review MSF Field Research Citation Viral Load Monitoring as a Tool to Reinforce Adherence: A Systematic Review Viral Load Monitoring as a Tool to Reinforce Adherence: A Systematic Review. J Acquir Immune Defic Syndr Publ Lippincott Williams Wilkins J J Acquir Immune Defic Syndr J Acquir Immune Defic Syndr Acquir Immune Defic Syndr [Internet]. 2013 [cited 2017 Jul 18];6464(1):74–874. Available from: http://hdl.handle.net/10144/336376

[8] National Zimbabwe Statistics Agency. Zimbabwe Population Census Results 2012 [Internet]. 2012 [cited 2017 Jul 13]. Available from: https://www.unicef.org/zimbabwe/Harare_Province.pdf

[9] HIV and AIDS in East and Southern Africa regional overview | AVERT [Internet]. [cited 2018 May 17]. Available from: https://www.avert.org/professionals/hiv-around-world/sub-saharan-africa/overview

[10] ICAP CU. Zimbabwe Population based HIV impact assessment 2015–2016 [Internet]. 2016 [cited 2017 Jul 13]. Available from: http://phia.icap.columbia.edu/wp-content/uploads/2016/11/ZIMBABWE-Factsheet.FIN_.pdf

[11] Davey DJ, Abrahams Z. Factors associated with unsuppressed viral load in HIV-1 infected patients on 1 st line antiretroviral therapy in South Africa Background. 2017 [cited 2018 Apr 18]; Available from: http://www.saaids.co.za/Presentations AIDS 2017/Wednesday, 14 June 2017/Hall 6/Dr Dvora Joseph Davey Unsuppressed VL PPT Final 30.5.pdf 10.1177/0956462417748859 PMC7053422 29334886

[12] Gupta RK, Gregson J, Parkin N, Haile-Selassie H, Tanuri A, Andrade Forero L, et al HIV-1 drug resistance before initiation or re-initiation of first-line antiretroviral therapy in low-income and middle-income countries: a systematic review and meta-regression analysis. Lancet Infect Dis [Internet]. 2018 3 1 [cited 2018 Apr 19];18(3):346–55. Available from: http://www.ncbi.nlm.nih.gov/pubmed/29198909 10.1016/S1473-3099(17)30702-8 29198909 PMC5835664

[13] Das M, Chu PL, Santos G-M, Scheer S, Vittinghoff E, McFarland W, et al Decreases in community viral load are accompanied by reductions in new HIV infections in San Francisco. PLoS One [Internet]. 2010 6 10 [cited 2018 May 16];5(6):e11068 Available from: http://www.ncbi.nlm.nih.gov/pubmed/20548786 10.1371/journal.pone.0011068 20548786 PMC2883572

[14] Bulage L, Ssewanyana I, Nankabirwa V, Nsubuga F, Kihembo C, Pande G, et al Factors Associated with Virological Non-suppression among HIV-Positive Patients on Antiretroviral Therapy in Uganda, August 2014–July 2015. BMC Infect Dis [Internet]. 2017 12 3 [cited 2017 Jul 13];17(1):326 Available from: http://bmcinfectdis.biomedcentral.com/articles/10.1186/s12879-017-2428-3 10.1186/s12879-017-2428-3 28468608 PMC5415758

[15] Fox MP, Berhanu R, Steegen K, Firnhaber C, Ive P, Spencer D, et al Intensive adherence counselling for HIV-infected individuals failing second-line antiretroviral therapy in Johannesburg, South Africa. Trop Med Int Heal [Internet]. 2016 9 1 [cited 2017 Jul 13];21(9):1131–7. Available from: http://doi.wiley.com/10.1111/tmi.12741 10.1111/tmi.12741 27383454

[16] Garone DB, Conradie K, Patten G, Cornell M, Frontières MS, Town C, et al High rate of virological re-suppression among patients failing second-line antiretroviral therapy following enhanced adherence support: A model of care in Khayelitsha, South Africa. South African Med J. 2013;14(4).

[17] MSF. MAKING VIRAL LOAD ROUTINE. Successes and challenges in the implementation of routine HIV viral load monitoring. PART 1: PROGRAMMATIC STRATEGIES (2016). [cited 2018 May 16]; Available from: https://aidsfree.usaid.gov/sites/default/files/making_vlprogstrat.pdf

